# Functional characterisation of PREL-1 and PREL-3 proteins as putative intramitochondrial phospholipid transporters governing homeostasis and organismal health in *Caenorhabditis elegans*

**DOI:** 10.3389/fmolb.2026.1892600

**Published:** 2026-08-19

**Authors:** Vijay Aditya Mavuduru, Lavanya Vadupu, Kiruthiga Shankar Kumar, Naga Bhushana Rao Karampudi, Writoban Basu Ball

**Affiliations:** 1 Mitochondrial Biogenesis Lab, Department of Biological Sciences, School of Engineering and Sciences, SRM University-AP, Amaravati, Andhra Pradesh, India; 2 Computational Biology and Bioinformatics Lab, Department of Biological Sciences, School of Engineering and Sciences, SRM University-AP, Amaravati, Andhra Pradesh, India

**Keywords:** ageing, *Caenorhabditis elegans*, cardiolipin, mitochondria, phosphatidylethanolamine, phospholipid transport, phospholipids

## Abstract

**Introduction:**

Mitochondrial phospholipids are crucial for maintaining structure and function; however, their roles and transport pathways are not fully understood in a multicellular model organism. In this study, we examined two putative *Caenorhabditis elegans* proteins, B0334.4 (PREL-1) and F15D3.6 (PREL-3), thought to be involved in intramitochondrial phospholipid transport.

**Methods:**

*In-silico* molecular docking analysis was performed to predict phospholipid binding specificity of PREL-1 and PREL-3. RNA interference (RNAi) was used to knockdown the expression of these proteins in *C. elegans.* Upon knockdown, development, lifespan, reproduction, locomotion, stress response, and mitochondrial phospholipid composition were analysed.

**Results:**

Docking studies predicted that phosphatidic acid (PA) preferentially binds to PREL-1 compared to PREL-3. Mitochondrial profiling revealed loss of mitochondrial phospholipid homeostasis upon RNAi knockdown. This resulted in mitochondrial dysfunction leading to impaired reproduction, respiration, locomotion, and elevated ROS levels in worms lacking these phospholipid transporters.

**Conclusion:**

Our findings support the role of PREL-1 and PREL-3 proteins in mitochondrial phospholipid homeostasis and suggest their function as PRELI- family phospholipid transporters. This study demonstrates the importance of these phospholipid transporters in mitochondrial function, growth, lifespan, and organism physiology.

## Graphical Abstract

**Figure:**
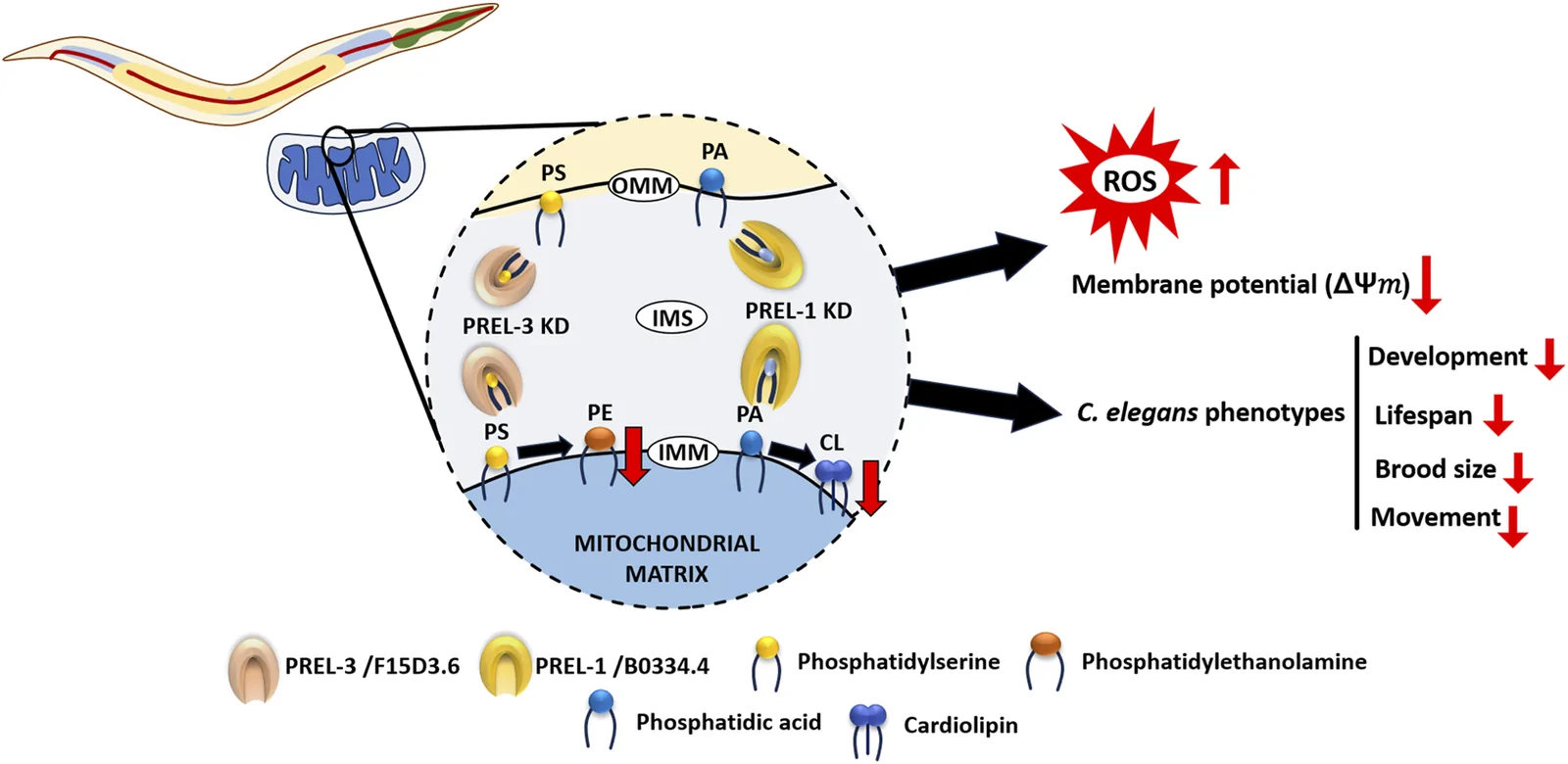
PREL-1 and PREL-3 mediate intramitochondrial phospholipid transport to maintain mitochondrial and organism health in *C. elegans*. Schematic model illustrating the role of PRELI- domain proteins B0334.4/PREL-1 and F15D3.6/PREL-3 in intramitochondrial phospholipid transport. RNAi-mediated knockdown (KD) of these proteins in *C. elegans* results in reduced mitochondrial membrane potential and increased reactive oxygen species (ROS). At the organismal level, disruption of phospholipid homeostasis impairs development, decreases life span, brood size, and locomotion in *C. elegans*, producing pleiotropic effects.

## 1 Introduction

Mitochondria are double-membrane-bound organelles that synthesise ATP, modulate intracellular signalling pathways, and maintain cellular homeostasis (Daum and Vance, 1997). The outer mitochondrial membrane (OMM) regulates metabolite exchange, apoptosis, mitochondrial dynamics and communication with other cell organelles while, the inner mitochondrial membrane (IMM) is the hub for electron transport chain (ETC) complexes and ATP synthase to synthesise ATP via oxidative phosphorylation (Kühlbrandt, 2015; Sogo and Yaffe, 1994; de Kroon et al., 1997).

In mitochondria, phospholipids support protein complex stabilisation in the membrane, membrane curvature, membrane dynamics, and also ATP production (Tasseva et al., 2013). Of all the phospholipids found in the mitochondria, phosphatidylcholine (PC), phosphatidylserine (PS), phosphatidylinositol (PI), and phosphatidylglycerol (PG) are bilayer-forming phospholipids that are synthesized in the endoplasmic reticulum (ER). Cardiolipin (CL) and phosphatidylethanolamine (PE)are unique non-bilayer-forming phospholipids of mitochondrial membranes that are synthesized in IMM from their precursors phosphatidic acid (PA)and PS, respectively (Basu Ball et al., 2018). PE is synthesised by the decarboxylation of PS by the enzymatic action of phosphatidylserine decarboxylase (encoded by PISD gene in humans) in the IMM. CL, a unique phospholipid found exclusively in the inner mitochondrial membrane, is synthesised through a series of enzymatic steps that begin with PA (Schlame and Greenberg, 2017). Therefore, to maintain the unique composition of mitochondrial membranes, PA and PS must be imported into the mitochondrial inner membrane. Since phospholipid precursors cannot freely diffuse across the aqueous intermembrane space, specialised lipid transport proteins are required to transfer them from the OMM to the IMM. The PRELI (*P*rotein of *R*elevant *E*volutionary and *L*ymphoid *I*nterest) domain is a highly conserved protein domain that plays a central role in phospholipid transport in eukaryotes. In *Saccharomyces cerevisiae*, lipid transport protein complexes like Ups1/Mdm35 and Ups2/Mdm35 are present in the intermembrane space (IMS), which help in the transport of PA and PS, respectively, to IMM (Connerth et al., 2012; Aaltonen et al., 2016; Miyata et al., 2016). In humans, PRELI family consists of PRELID1, PRELID2, and PRELID3A/B. These proteins interact with TRAIP1, functional ortholog of Mdm35 (present in *Saccharomyces cerevisiae*), and function as phospholipid transporters. Hence, phospholipid homeostasis is regulated through these phospholipid biosynthesis, transport, and degradation pathways (Carman and Han, 2009). Disruption of these phospholipid transporters is known to destabilise mitochondrial membranes and impair cellular respiration. Altered phospholipid composition has recently been identified as a causative factor in several human disorders (Joshi et al., 2023). For example, mutations in the TAZ (tafazzin) gene affect CL remodelling and cause Barth syndrome (Clarke et al., 2013), whereas phosphatidylserine decarboxylase (PISD) deficiency results in depleted mitochondrial PE levels and leads to Liberfarb syndrome (Peter et al., 2019). TAMM41, a phosphatidate cytidylyltransferase, is an important biosynthetic enzyme in the mitochondrial CL biosynthesis pathway (Blunsom et al., 2018). Biallelic variants in TAMM41 are associated with low muscle cardiolipin levels, leading to neonatal mitochondrial disease (Thompson et al., 2022). Mitochondrial dysfunction is also known to be associated with cancer, metabolic and cardiovascular diseases in humans (Zhunina et al., 2021). Despite the vital roles of mitochondrial phospholipid homeostasis in human health and disease, the effects of disrupting these mitochondrial phospholipid transport proteins in multicellular organisms remain poorly explored.

*Caenorhabditis elegans* is a suitable model organism for studying phospholipid regulation and its impact on mitochondrial function because of its ease of studying development, lifespan, and health (Campbell and Zuryn, 2024). With a transparent body, a convenient lifespan, and the availability of GFP-tagged reporter strains, *C. elegans* allows the estimation of mitochondrial dysfunction at the organism level. However, phospholipid studies in *C. elegans* are limited by the organism’s small size, which makes it difficult to obtain sufficient material for mitochondrial profiling by conventional biochemical methods. *C. elegans* also contains high levels of polyunsaturated fatty acids (PUFAs), which are highly susceptible to oxidative degradation during extraction, necessitating proper preventive measures (Penkov et al., 2026). These limitations might have hindered the study of phospholipid metabolism in this model organism.

In this study, we aimed to understand the roles of two putative intramitochondrial phospholipid transporter proteins in *C. elegans.* We found that these two proteins, B0334.4 (PREL-1) and F15D3.6 (PREL-3), were involved in maintaining phospholipid homeostasis. By combining *in silico* analyses, RNAi-mediated knockdown, physiological assays, and optimised mitochondrial phospholipid profiling, we investigated the effects of loss of these proteins on mitochondrial function, development, and ageing in a multicellular model organism.

## 2 Materials and methods

### 2.1 *In-silico* structural and molecular docking analysis

PRELI-family homologous protein sequences in *C. elegans* were identified using BLASTP searches from UniProtKB/Swiss-Prot database from the NCBI BLAST + suite3, combined with custom scripts that accessed the UniProt REST API(Bairoch and Apweiler, 2000; Camacho et al., 2009; UniProt Consortium, 2025). Iterative searches were performed using multiple amino acid substitution matrices, including BLOSUM45, BLOSUM62, BLOSUM80, PAM30, PAM70, and PAM250, to improve detection of both conserved and distantly related PRELI-family homologs (Henikoff and Henikoff, 1992). All searches used an E-value cutoff of 1 × 10^−5^, default scoring parameters, and low-complexity filtering. The sequences retrieved were manually curated. The redundant entries, incomplete fragments, and low-confidence annotations were removed before downstream analyses.

Multiple sequence alignment was performed using MAFFT v7.505 with the “-auto” flag (Katoh and Standley, 2013). This option picks the L-INS-i iterative refinement strategy based on the dataset’s sequence similarity and complexity. The algorithm applies local pairwise alignment and multiple refinement cycles to place residues accurately within the conserved PRELI domain.

PREL-1-associated and PREL-3-associated homologs were aligned separately, then merged into a single PREL-family alignment for phylogenetic reconstruction and comparative modelling. Phylogenetic gene treewas constructed using FastTree v2.1.11 under approximate maximum-likelihood conditions with default amino acid models (Price et al., 2010). It was then midpoint-rooted and visualized using the ETE3 Python framework together with Biopython Phylo modules.

Homology modelling requires structural templates with sufficient evolutionary and structural similarity to the target proteins. To identify suitable modelling templates for *C. elegans* PRELI-family proteins, we searched the Protein Data Bank (PDB) based on experimentally determined crystal structures, conservation of the PRELI lipid-transfer domain, sequence identity, and similarity relative to the target proteins based on the MSA results obtained. For modelling of PREL-1 the human PRELID1–TRIAP1 complex structure (PDB: 6I3V) was selected as the primary template as it shares 35.9% identity and 75.0% similarity, whereas the human PRELID3B structure (PDB: 6I4Y) was used for modelling of PREL-3 as it shares 40.6% identity and 66.3% similarity with *C. elegans prel-3* sequence (Miliara et al., 2019) (Table 1). Template structures were processed using custom Python scripts implemented with Biopython structural analysis modules. Non-target chains, solvent molecules, heteroatoms, affinity tags, and crystallographic artifacts were removed before modelling, and chain B corresponding to the PRELI-domain region was retained from both template structures.

**TABLE 1:** Structural templates selected for comparative modelling of *Caenorhabditis elegans*PREL-1 and PREL-3 proteins.

S.No	Query	Template	PDB id	Identity %	Similarity %	Resolution Å	Coverage %
1.	*prel-1*	PRELID1_HUMAN	6I3V	35.9%	75.0%	1.9 Å	82.4%
2.	*prel-3*	PRELID3B_HUMAN	6I4Y	40.6%	66.3%	2.0 Å	82.8%

Three-dimensional structural models of *C. elegans* PREL-1 and PREL-3 proteins were generated using MODELLER v10.8 with the AutoModel module (Webb and Sali, 2016). Structural modelling utilized cleaned template coordinates corresponding to chain B from PDB structure 6I3V for PREL-1 modelling and chain B from PDB structure 6I4Y for PREL-3 modelling. Five independent models were generated for each target protein using variable target function method (VTFM) optimization together with restrained molecular dynamics refinement under default AutoModel parameters. We used normalized Discrete Optimized Protein Energy (DOPE) scoring together with template-versus-model Cα root mean square deviation (RMSD) calculations for assessing the quality of the structure generated. Structural quality assessment was performed using normalized DOPE scoring together with stereochemical evaluation by Ramachandran analysis. Stereochemical assessment was subsequently performed using custom Python-based Ramachandran analysis scripts implemented with Biopython and Matplotlib libraries. Backbone φ and ψ dihedral angles were calculated for all residues within the final representative structures and classified into favored, additionally allowed, and outlier conformational regions.

The generated structures represent comparative computational models intended primarily for structural and evolutionary interpretation rather than experimentally validated atomic-resolution conformations.

#### 2.1.1 Molecular docking

Phosphatidic acid (PA) ligand DOPA species with CID 954716 was obtained from the PubChem database and converted into MOL2 format and validated using Open Babel v3.1.1 with default calculations (O’Boyle et al., 2011). DOPA is chosen due to its less complex nature, containing smaller fatty acid chains compared to other PA species. Default GNINA/Open Babel protonation and atom-typing procedures were retained during docking preparation workflows. Flexible ligand conformations were retained during preprocessing to permit torsional sampling during docking simulations.

Docking simulations were performed using GNINA v1.3 (McNutt et al., 2025) (master build 97fa6bc+, compiled 3 October 2024), a convolutional neural network-enhanced docking framework derived from AutoDock Vina (Eberhardt et al., 2021) and smina (Koes et al., 2013). Receptor structures were prepared using PyMOL v2.5.017 following removal of solvent molecules and non-biological heteroatoms. Docking regions were selected based on conserved PRELI-domain cavity architecture identified through comparative structural alignment with experimentally resolved PRELI-domain phospholipid-transfer proteins together with inspection of conserved hydrophobic channel residues.

Docking grids were centered at coordinates x = −46.904, y = 46.207, and z = −13.530 with search-space dimensions of 25 × 25 × 25 Å to give preferential selection for binding target/pocket which is known to pack the ligand. Docking calculations were performed using an exhaustiveness parameter of 32 with a maximum output of 20 docking poses per receptor–ligand system. An exhaustiveness value of 32 was selected to improve conformational sampling robustness relative to default GNINA search settings.

During molecular docking, ligands were treated flexibly for conformational sampling, while receptors were kept rigid. Rigid-receptor docking was chosen to standardize conditions and allow direct structural comparisons of the conserved PRELI-domain phospholipid-binding pockets across the homologous models. GNINA’s default convolutional neural network (CNN) rescoring was utilized to calculate empirical Vina binding affinities alongside deep learning-based pose quality predictions. Output metrics included predicted binding affinities, intramolecular energies, and CNN scores for each generated conformation. CNN pose scores (Table 2) were interpreted as measures of docking-pose plausibility, whereas CNN affinity values represented machine learning–based estimates of binding strength. Replicate docking poses generated with identical parameters consistently localized within the predicted PRELI-domain cavity. Representative poses were selected based on high CNN pose-quality scores (Table 2), consistent ligand orientation, and favourable binding affinities, and were subsequently converted to PDB format for visualization and interaction analysis. We used Protein-Ligand Interaction Profiler (PLIP) v3.0.018 (Salentin et al., 2015) for Protein Ligand Interaction Analysis. All computational analyses were performed on an NVIDIA GeForce RTX 3060 GPU running Debian Linux 12.10 (64-bit).

**TABLE 2:** GNINA-based molecular docking analysis of PREL-family protein–phospholipid interactions.

S.No	Complex	Best binding affinity (kcal/mol)	Affinity range (Top 20 poses)	Highest CNN pose score	Highest CNN affinity
1.	*PREL*-1-PA	−5.83	−4.92 to −5.83	0.63	5.30
2.	*PREL*-3-PA	−3.80	−3.19 to −3.80	0.66	4.92
3.	*PREL-3-PS*	−4.90	−4.40 to −4.90	0.44	6.26

### 2.2 Strains and media

*C. elegans* strains used in this study, Bristol (N2), and *daf*-16::GFP were obtained from the *Caenorhabditis* Genome Centre (CGC). Strains were grown and maintained on NGM plates at 20 °C (Avery, 1993).

### 2.3 RNA interference

RNAi strains used for this study are HT115 (empty vector control - the HT115 *E. coli* strain carrying the empty vector plasmid pPD129.36 (L4440) (Conte et al., 2015), B0334.4/*prel-*1 RNAi (clone, Ahringer Library) and F15D3.6/*prel-3* RNAi (clone, Ahringer Library). For RNAi-mediated knockdown experiments, bacterial cultures harbouring the specific clones were grown overnight at 37 °C in LB medium containing 50 μg/mL ampicillin. The following day, cultures were spun down and resuspended in 1/10 of the original volume containing 50 μg/mL ampicillin and induced with 0.5 mM IPTG. Cultures were seeded onto NGM agar plates containing 50 μg/mL carbenicillin and 1 mM IPTG. Protein level downregulation of expression could not be validated because of the unavailability of specific antibodies. We quantified the mRNA level downregulation using quantitative PCR.

### 2.4 Lifespan assays

All lifespan assays were performed at 20 °C. Animals were age-synchronised and allowed to develop on control empty vector RNAi plates at 20 °C. 132 Young adults were transferred to 6 small 35 mm experimental RNAi plates containing 1 mM IPTG, 50 μg/mL carbenicillin, and 50 µM FUDR to prevent larval hatching. Animals were examined every alternate day until the end of the assay. Worms that did not show any movement to a gentle prick were scored as dead. Worms crawling out of the plate or showing exploded organs are considered censored. Kaplan–Meier survival analysis of *C. elegans* treated with *prel-*1 RNAi and *prel-*3 RNAi was compared with control worms using the Log-Rank test, with a Bonferroni-corrected p-value of <0.0001. Survival data were analysed using OASIS2 (Online Application for Survival Analysis of Lifespan Assays) (Han et al., 2016). The experiments were performed in triplicate, and p-values were determined from a Chi-square (χ2) test.

### 2.5 Brood size

Hermaphrodites were allowed to lay eggs on HT115 (empty vector control). L1 worms were age-synchronised and then transferred to plates containing empty vector control, *prel-*1 RNAi, and *prel-*3 RNAi. The worms were transferred to new plates every day until egg-laying ceased, and plates with eggs were counted. The total number of progenies was counted (Hodgkin and Barnes, 1991). The differences between wildtype and mutant worms were determined using one-way ANOVA, with Dunnett’s *post hoc* test.

### 2.6 Developmental analysis

L1 worms were fed empty vector control (Ht115), *prel-1* RNAi, and *prel-3* RNAi until they reached the L4 stage. Worms were imaged every 12 h to track their development. Images were taken using an Optika brightfield microscope at ×10 magnification. Body sizes were measured using ImageJ and difference in body sizes between control and mutant groups were determined using Two-way ANOVA, with Tukey *post hoc* analysis.

### 2.7 Ethanolamine supplementation and rescue

NGM-IPTG-carbencillin plates supplemented with 25 mM ethanolamine were prepared and seeded with *prel-1* and *prel-3* RNAi bacterial cultures. Synchronized L1 worms were transferred onto the RNAi plates containing ethanolamine. Control worms were maintained on RNAi plates without ethanolamine. Worms were imaged every 12 h till the control worms reached young adult stage. Body sizes were calculated using ImageJ and analysed using two way ANOVA with Tukey’s multiple comparisons *post hoc* correction.

### 2.8 DAF-16 nuclear localization

Age-synchronised young adult worms *daf-16*::GFP are transferred to RNAi plates. After 3 days, worms were washed thrice with M9 and placed on 2% agarose pads, anaesthetised with 5 μL of 20 mM Levamisole in M9 buffer. GFP intensity was scored in the young adult worms using an Optika epifluorescent microscope at 10X magnification using a GFP filter set. Images were acquired using constant exposure time and intensity. Around 30 worms were analysed per condition. Experiment was conducted using at least three independent biological replicates. The worms were blind scored into cytoplasmic, intermediate and nuclear categories and subsequently, graphs were plotted using GraphPad.

### 2.9 Tail thrashing assay

The thrashing activity of RNAi treated worms was measured on day 1, day 5, and day 8 of adulthood by placing a single worm in a drop of M9 buffer on a glass slide. The thrashes were recorded for 20 s using an Optika microscope in brightfield. The rate of thrashing was measured for 15 worms per condition. The experiment was performed in triplicate, and p-values were determined using two way ANOVA with Tukey *post hoc* correction.

### 2.10 RNA isolation and quantitative PCR

For RNA preparation, 500 worms were grown at 20 °C on empty vector (Ht115) till L4 stage and are transferred to RNAi plates for 3 days. qPCR runs were performed following established protocol (Yanase, 2020). Briefly, worms were washed 4 times in M9 buffer to remove bacteria. Worms were lysed using liquid nitrogen, and RNA was extracted using the RNeasy Mini kit (QIAGEN) following the manufacturer’s protocol. RNA concentration and quality were assessed using a Nanophotometer. 1 μg of RNA was used to synthesise cDNA with reverse transcriptase (iScriptTM cDNA synthesis kit, Bio-Rad). Samples were analysed by the 2^−ΔΔCT^ method. Quantified via quantitative PCR using gene-specific primers for the mitochondrial phospholipid transporters:*prel-1* (Forward 5′-AATCATCCGAGGATAATCAGCA-3′, Reverse 5′-CACAATCGATTGAAACTGTGAC-3′) and *prel-3* (Forward 5′-TTACACTTCCAGCATTCGC-3′, Reverse 5′-CCTTGTCGACCCTTATTAGC-3′), with act-1 (Forward 5′-GGAGTCATGGTCGGTATGG-3′ and, Reverse 5′-CTTGAGGGTAAGGATACCTCTC-3′) serving as the internal reference control. The experiment was performed in triplicate, and p-values were determined from a one-way ANOVA, Dunnett’s *post hoc* test.

### 2.11 Measurement of oxygen consumption rate (OCR) in *Caenorhabditis elegans*

Oxygen consumption rate was measured using the Hansatech DW2/2 Liquid-Phase Oxygen Electrode Chamber. Following 3 days of RNAi-mediated adult inactivation, worms were washed thrice from the plate with M9 buffer. Worms were diluted with M9 buffer, and 10 µL aliquots were used for counting the number of worms. 5,000 worms were used for measuring respiration in each experiment. RNAi was administered at L4 or young adult stage to measure OCR in wildtype and mutant worms to avoid any effects of developmental defects observed in the larval stages (L1 worms). Worm populations were age- and stage-matched (day 1 adults) across all conditions prior to measurement. Briefly, 5,000 worms were added to the oxytherm chamber, in a final volume of 1 mL of M9 buffer. The oxygen consumption rate was captured, and the data were analysed using OxyTrace + software. The experiment was performed in triplicate, and p-values were determined using the one-way ANOVA.

### 2.12 Measurement of reactive oxygen species (ROS)

Reactive oxygen species (ROS) were measured using 2′,7′-dichlorodihydrofluorescein diacetate (H2DCFDA) (Yoon et al., 2018). H2DCFDA is enzymatically deacetylated to H2DCF, which, upon exposure to ROS, rapidly oxidises to DCF. 100 synchronised worms per condition subjected to adult RNAI inactivation for 3 days were used to measure ROS levels. H2O2 was used as a positive control for detecting ROS species. ROS levels were assessed using a TECAN multimode plate reader, with measurements taken at 1-h intervals for a total of 5 h. The data was normalized with no DCF and no worm controls post assay. The experiment was performed in triplicate with technical replicates, and p-values were determined using two-way ANOVA with Tukey *post hoc* analysis.

### 2.13 Mitochondrial isolation

Following 3 days of RNAi-mediated adult inactivation, 200,000 worms were washed once with M9 buffer and then collected for mitochondrial isolation. After collection, worms were incubated in 20 mL of fresh M9 buffer on a rotary shaker for 30 min to remove bacteria. The worms were washed twice with M9, centrifuged at 1,000 g, and the worm pellet was resuspended in 1 mL of ice-cold mitochondrial isolation buffer (1 M sucrose, 0.1 M Tris/MOPS, 0.1 M EDTA/Tris, and 1 mM PMSF). The contents were then transferred to a pre-chilled Dounce homogeniser, and worms were disrupted by homogenization. The homogenate was then centrifuged at 2000 g for 5 min at 4 °C. The supernatant was transferred to a fresh tube and centrifuged at 13,000 g for 20 min at 4 °C using a Kubota high-speed centrifuge. The supernatant was discarded, and the pellet containing mitochondria was resuspended in 100 μL of mitochondrial isolation buffer (250 mM sucrose, 10 mM MOPS-KOH, pH 7.2, 1 mM EDTA). The mitochondrial sample was stored at −80 °C until further use. Mitochondrial protein concentration was determined using the Bradford assay (Grad et al., 2007).

### 2.14 Phospholipid isolation and thin-layer chromatography (TLC)

Phospholipids were extracted using an equal amount of mitochondrial protein (200 µg) using the Folch method (Folch et al., 1957). All the extraction steps were carried out on ice. Phospholipids were separated using a mobile phase containing chloroform/ethanol/water/triethylamine (30:35:7:35) solvent system, and using TLC silica gel 60 plates as the stationary phase (Sigma-Aldrich). TLC plates were activated by treating with 1.8% boric acid at 110 °C. The TLC chamber was equilibrated using the solvent system for 30 min. Phospholipids were spotted on TLC plates and dried. Phospholipid standards obtained from Avanti Polar Lipids were used as markers for individual phospholipids. After the TLC run, the plate was air-dried for 1 h, then stained with a CuSO4 solution containing 10% orthophosphoric acid. The plate was dried and placed at 180 °C for 5 min. Dark phospholipid bands were visualised and quantified using ImageJ. Experiments were carried in triplicates, and graphs were plotted using GraphPad.

## 3 Results

### 3.1 Sequence analysis, homology modelling and structural validation of PRELI-family proteins

**FIGURE 1:**
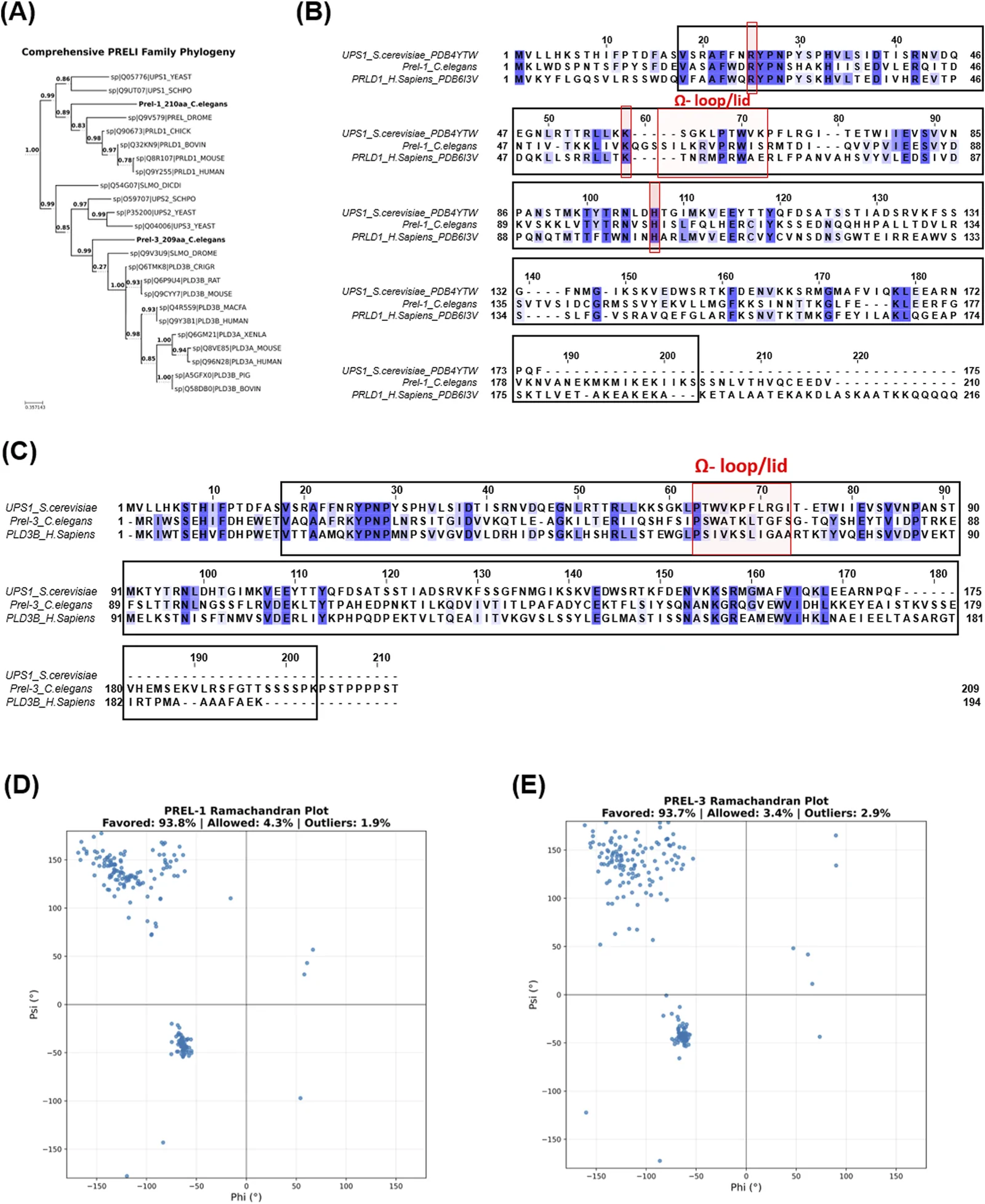
Comparative sequence and phylogenetic analysis of C. elegans PREL-1 and PREL-3 with PRELI-domain family homologues for structural template selection. **(A)** Phylogenetic tree of the curated dataset containing PRELID1-, PRELID3-, UPS1-, UPS2-, UPS3-, and slowmo-like proteins from *Homo sapiens, Mus musculus, Bos taurus, Rattus norvegicus, Drosophila melanogaster, Schizosaccharomyces pombe, Saccharomyces cerevisiae, Dictyostelium discoideum, Xenopus laevis, Gallus gallus, and Caenorhabditis*elegans was midpoint-rooted using ETE3 for visualization. The tree was constructed using aligned PRELI-domain sequences, with sequence names corresponding to their respective species and protein identifiers. Branch lengths represent the estimated number of amino acid substitutions per site, as indicated by the scale bar. Numerical values shown at internal nodes correspond to FastTree branch-support values. **(B)** Multiple sequence alignment of *C. elegans* PREL-1 with experimentally characterized PRELI-domain homologues used for comparative structural analysis. Conserved regions within the PRELI lipid-binding domain are highlighted in a box, including residues contributing to the predicted phospholipid-binding cavity are highlighted in red colour. **(C)** Multiple sequence alignment of *C. elegans* PREL-3 with PRELI-family homologues and experimentally resolved structural templates. Conserved regions within the PRELI lipid-binding domain are highlighted in a box, including residues contributing to the predicted phospholipid-binding cavity are highlighted in red colour. **(D)** Ramachandran plots validating PREL-1 model. The stereochemical quality of the predicted 3D structures of *C. elegans* PREL-1. The majority of residues in both models fall within the favoured 93.8% and allowed 4.3% regions, with only 1.9% outliers, respectively. These indicate reliable structural predictions suitable for docking studies. **(E)** Ramachandran plots validating PREL-3 model. The stereochemical quality of the predicted 3D structures of *C. elegans* PREL-1. The majority of residues in both models fall within the favoured 93.7% and allowed 3.4% regions, with only 2.9% outliers, respectively. These indicate reliable structural predictions suitable for docking studies.

A total of 24 preliminary PRELI-family homologous sequences were retrieved through iterative BLASTP searches using multiple substitution matrices. After removal of redundant and low-confidence entries, 22 non-redundant homologs were retained for comparative analysis and phylogenetic reconstruction (Figure 1A). The curated dataset included PRELID1-, PRELID3-, UPS1-, UPS2-, UPS3-, and slowmo-like proteins from *Homo sapiens, Mus musculus, Bos taurus, Rattus norvegicus, Drosophila melanogaster, Schizosaccharomyces pombe, Saccharomyces cerevisiae, Dictyostelium discoideum, Xenopus laevis, Gallus gallus, and Caenorhabditis elegans.*

Pairwise sequence identity and similarity analyses were performed to further evaluate the evolutionary relationship of *C. elegans* PREL-1 and PREL-3 with representative PRELI-family homologues. The heatmap analyses demonstrated that both PREL proteins retain measurable sequence conservation with human and yeast PRELI-domain proteins, supporting conservation of the PRELI-family architecture (Supplementary Figures 1A,B, 2A,B). For PREL-1 and PREL-3, sequence similarity analysis showed conservation with PRELI homologues across different substitution matrices, indicating preservation of amino acid physicochemical properties despite sequence divergence with higher similarity compared with identity values, suggesting that functionally relevant amino acid properties are maintained despite evolutionary variation (Supplementary Figures 2A,B). The conserved regions were mainly associated with the PRELI-domain core, whereas greater sequence divergence was observed in variable terminal and loop regions. Conservation of residues contributing to the lipid-binding cavity among *C. elegans*, human, and yeast homologues supports preservation of the characteristic PRELI-domain phospholipid-binding architecture.

The PREL-1 modelling alignment contained three sequences comprising *C. elegans* PREL-1 together with homologous experimentally resolved PRELI-domain templates from human PRELID1 (PDB: 6I3V) and yeast UPS1 (PDB: 4YTW) (Figure 1B). The PRELID3 modelling alignment contained three sequences including *C. elegans* PREL-3 together with PRELID1, and UPS1 homologous template structures depicted in (Figure 1C).

Experimentally resolved PRELI-domain structures from the Protein Data Bank (PDB) were evaluated based on sequence identity, similarity, structural coverage, resolution, and conservation of the PRELI lipid-binding domain depicted in Supplementary Figures 1A,B, 2A,B. Human PRELID1 (PDB ID: 6I3V) and PRELID3B (PDB ID: 6I4Y) were selected as modelling templates because they provided the optimal combination of PRELI-domain conservation, structural coverage, and experimentally determined high-resolution structures.

### 3.2 Structural validation of comparative models

Structural validation demonstrated favourable quality for the selected representative models used in subsequent docking analyses. The PREL-1 model exhibited an RMSD of 4.219 Å and a DOPE score of −13,713.543, whereas the PREL-3 model exhibited an RMSD of 3.310 Å and a DOPE score of −13,482.933. Together with the Ramachandran plot assessment these results supported the suitability of both models for downstream structural and docking analyses. Ramachandran plot analysis of the selected PREL-1 model (Figure 1D), revealed that approximately 93.75% of residues occupied favoured conformational regions, 4.33% occupied additionally allowed regions, and only 1.92% were classified as outliers. We observed that PREL -3 had 93.72% favoured residues, 3.38% allowed residues, and 2.90% outliers which indicates that both the models are suitable for downstream docking (Figure 1E).

### 3.3 Molecular docking and protein–ligand interaction analysis of phosphatidic acid binding

**FIGURE 2:**
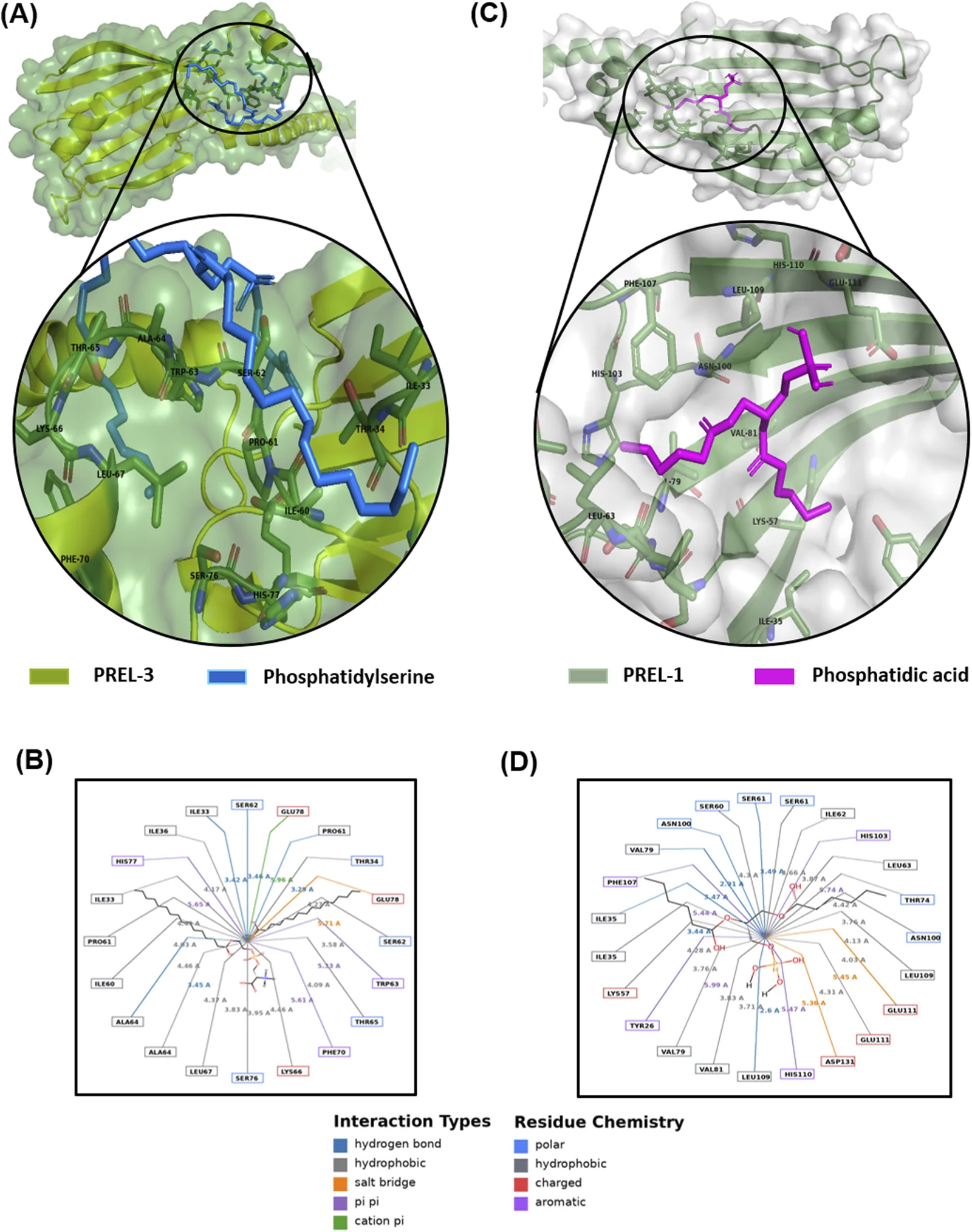
Comparative molecular docking and interaction analysis of PRELI-domain proteins with phosphatidylserine (PS) and phosphatidic acid (PA). **(A)** Predicted docking conformation of phosphatidylserine (PS) within the conserved PRELI-domain lipid-binding cavity of the *C. elegans* PREL-3 structural model. PREL-3 is represented using cartoon and molecular surface visualization (green), while PS is shown as a blue stick representation. The magnified binding-pocket view highlights the ligand orientation and surrounding amino acid residues predicted to contribute to phospholipid stabilization. **(B)** Two-dimensional protein–ligand interaction map of the PREL-3–PS docking complex showing residues involved in ligand recognition. The interaction network illustrates predicted hydrogen bonds, hydrophobic contacts, salt bridges, π–π interactions, and cation–π interactions. Residue colours indicate biochemical properties, including polar, hydrophobic, charged, and aromatic residues. The displayed interaction distances represent residue–ligand contact distances in Å.**(C)** Predicted docking conformation of phosphatidic acid (PA) within the PRELI-domain cavity of the C. elegans PREL-1 structural model. PREL-1 is represented as a cartoon structure with transparent surface visualization, and PA is shown as a magenta stick representation. The enlarged region shows PA positioning within the predicted lipid-binding pocket and neighbouring interacting residues. **(D)** Two-dimensional protein–ligand interaction map of the PREL-1–PA docking complex illustrating amino acid residues contributing to ligand stabilization through hydrophobic, hydrogen-bonding, electrostatic, and aromatic interactions. Interaction types and residue chemical classifications are indicated according to the colour scheme shown below the interaction maps.

To investigate the potential phospholipid-binding characteristics of PRELI-family proteins, molecular docking simulations were performed using phosphatidic acid (PA) with the modelled PREL-1 and PREL-3 structures. Additionally, phosphatidylserine (PS) docking was performed with PREL-3 to evaluate its potential interaction with the PE precursor phospholipid (Figure 2A). Docking analyses were interpreted as computational predictions of lipid-binding interactions and not as direct measurements of phospholipid transport activity.

GNINA-based docking predicted a more favourable PA-binding score for PREL-1 compared with PREL-3. The PREL-1–PA complex displayed predicted docking affinities ranging from −4.92 to −5.83 kcal/mol, with the best-scoring pose exhibiting a predicted affinity of −5.83 kcal/mol (Table 2) (Figure 2C). In comparison, the PREL-3–PA complex showed weaker predicted docking affinities ranging from −3.19 to −3.80 kcal/mol depicted in Supplementary Figure 3A. Since, mitochondrial phosphatidylserine serves as the precursor for phosphatidylethanolamine (PE) synthesis, PREL-3–PS docking was additionally performed. The PREL-3–PS complex showed a predicted binding affinity of −4.90 kcal/mol and formed interactions within the PRELI-domain cavity (Figure 2B). These results suggest that PREL-3 may accommodate PS within its lipid-binding pocket, supporting a possible association with mitochondrial PE homeostasis. However, experimental lipid-transfer assays are required to confirm direct substrate specificity. These values represent computational docking scores and should not be interpreted as experimentally determined binding constants.

Protein–ligand interaction analysis of the PREL-1–PA complex revealed several residues contributing to ligand stabilization within the conserved PRELI-domain cavity, including Ser60, Ser61, Lys57, Asp131, His103, Glu111, Tyr26, Phe107, and Asn100. These residues participated in a combination of hydrogen bonding, hydrophobic contacts, salt bridges, and aromatic interactions, resulting in an extensive predicted interaction network (Figure 2D).

In comparison, the PREL-3–PA docking complex exhibited a comparatively smaller interaction network involving residues including Ser62, Trp63, Ala64, Lys66, Leu67, Thr68, Ile60, Pro61, and Ile33 through hydrophobic interactions, hydrogen bonding, salt bridge formation, and aromatic contacts (Supplementary Figure 3B). The residues discussed represent major interactions identified from PLIP analysis based on interaction type and contribution to ligand stabilization; therefore, not all displayed residue contacts are individually discussed.

Protein–ligand interaction analysis of the PREL-3–PS complex revealed several residues contributing to phosphatidylserine stabilization within the conserved PRELI-domain cavity, including Ser62, Ser76, Lys66, Glu78, His77, Trp63, Thr34, Thr65, Thr68, Ile33, Ile36, Ala64, Leu67, Pro61, and Phe70. These residues participated in a combination of hydrogen bonding, hydrophobic contacts, salt bridge formation, and aromatic interactions, supporting the predicted accommodation of PS within the PREL-3 lipid-binding pocket (Figure 2B).

Collectively, the docking analyses suggest that PREL-1 forms a more favourable predicted interaction profile with PA under the computational conditions tested, whereas PREL-3 demonstrates potential interaction with PS. These findings provide structural hypotheses for PRELI-family phospholipid recognition but do not independently establish lipid transport activity.

Predicted binding affinities and convolutional neural network (CNN)-based docking scores were obtained from GNINA docking simulations for PREL-1 and PREL-3 phospholipid complexes. Binding affinity values are reported as predicted docking energies (kcal/mol) and do not represent experimentally measured binding constants. The affinity range represents the distribution of the top 20 generated docking conformations. CNN pose scores indicate the predicted reliability of ligand binding poses, whereas CNN affinity scores represent deep-learning-based estimates of relative ligand-binding strength.

### 3.4 Intramitochondrial phospholipid transporters regulate development and maintain lifespan in *Caenorhabditis elegans*

**FIGURE 3:**
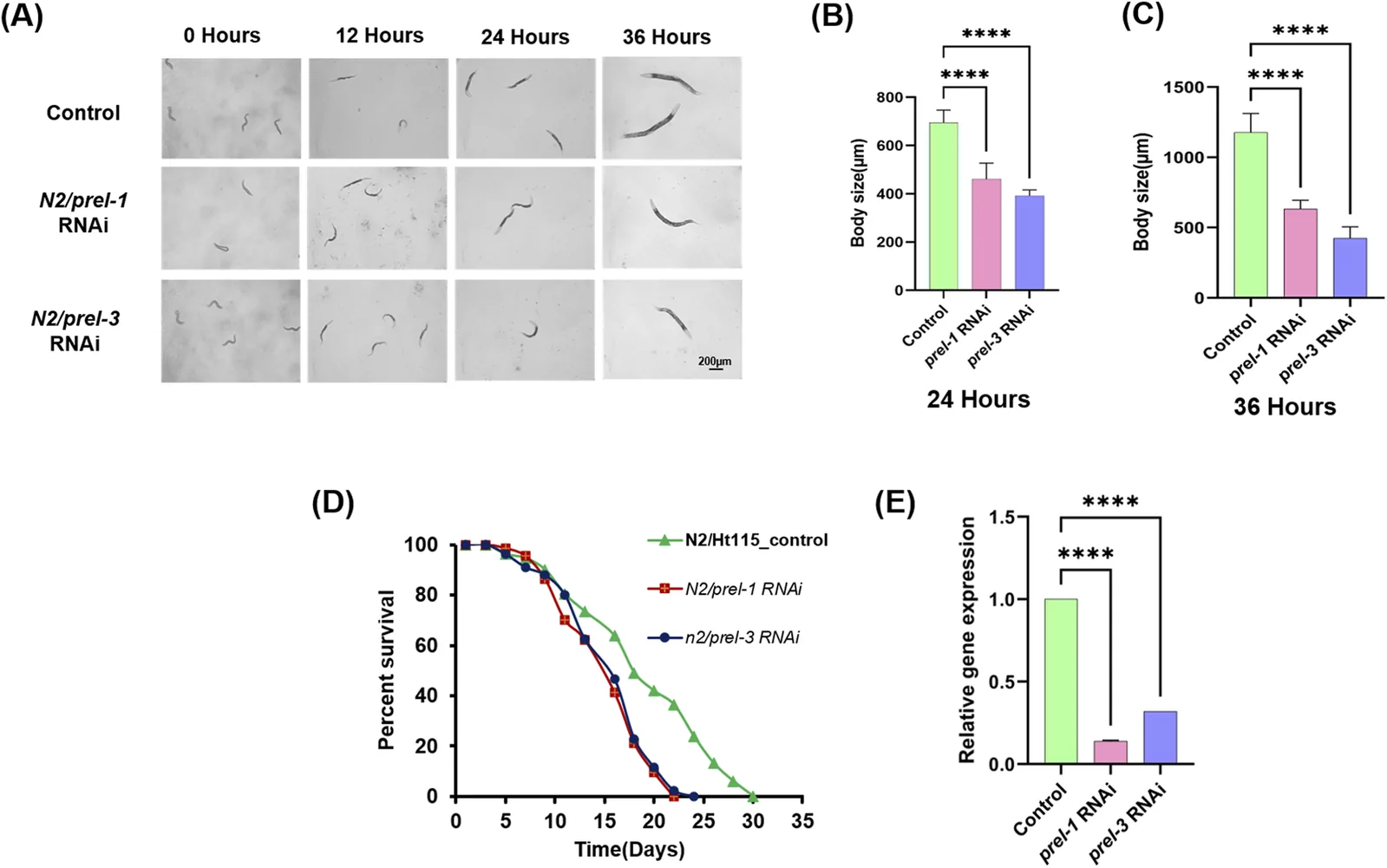
PREL-1 and PREL-3 are essential for the development and longevity of *Caenorhabditis elegans*. **(A)** N2 worms were grown on control RNAi(Ht115), *prel-1* RNAi, and *prel-3* RNAi bacteria. Worms were imaged at 0, 12, 24, and 36 h post-hatching to monitor developmental progression. Scale bar = 200 µm. **(B)** Quantification of body size (µm) at 24 h in control, *prel-1* RNAi, and *prel-3* RNAi worms. Data are presented as mean ± SEM (****p < 0.0001, one-way ANOVA). **(C)** Quantification of body size (µm) at 36 h in control, *prel-1* RNAi, and *prel-3* RNAi worms. Data are presented as mean ± SEM (****p < 0.0001, one-way ANOVA). **(D)** Lifespan analysis of N2/HT115 control, N2/*prel-1* RNAi, and N2/*prel-3* RNAi worms (n = 132 worms per condition). Survival data were analysed using OASIS2 (Online Application for Survival Analysis of Lifespan Assays). Kaplan–Meier survival curves were generated, and statistical significance was determined using the Log-Rank test with Bonferroni post-hoc correction (p < 0.0001). **(E)** Quantitative RT-PCR showing relative mRNA expression of *prel-1* and *prel-3* following RNAi knockdown, normalized to act-1 as a housekeeping gene. Data are presented as mean ± SEM (****p < 0.0001, one-way ANOVA).

To determine the physiological relevance of PREL-1 and PREL-3 we analysed the effects of RNAi-mediated knockdown of these genes on the organismal-level development and ageing of *C. elegans*. Knockdown worms exhibited a pronounced delay in larval development as compared to control worms (Figure 3A). Body lengths of knockdown worms at 24 h and 36 h of development showed decreased body sizes in PREL-1 (p value <0.0001) and PREL-3 (p value < 0.0001) knockdown worms (Figures 3B,C). This phenotype is consistent with previous observations linking mitochondrial lipid imbalance to impaired growth and developmental progression in varied organisms (Sergi, 2025). Ethanolamine (Etn) supplementation significantly rescued the developmental delay caused by prel-3 RNAi knockdown, restoring body size to near-control levels at both 24 h (p value < 0.0001) and 36 h (p value < 0.0001) post-synchronization (Supplementary Figure 4). In contrast, ethanolamine supplementation only partially rescued the delay caused by *prel-1* RNAi at 24 h (p value = 0.0108) (Supplementary Figures 4A,B), and this rescue was no longer significant by 36 h (p value = 0.9816) (Supplementary Figure 4D). These results indicate that the developmental defect in prel-3 knockdown animals is more directly linked to ethanolamine deficiency than that in prel-1 knockdown animals. We next assessed whether disruption of intramitochondrial phospholipid transport influences lifespan in adult worms (adult inactivation). Kaplan–Meier survival analysis revealed a statistically significant reduction in lifespan in both knockdown conditions (Log-Rank, Bonferroni-corrected p < 0.0001). Control worms displayed a mean lifespan of approximately 19.1 days, whereas PREL-1 and PREL-3-depleted animals survived for approximately 15.31 and 15.64 days, respectively (Figure 3D). mRNA level expression in the *prel*-1 or *prel-3* worms were analysed to validate knocking studies and knockdown showed ∼85% reduction in *prel-1* and ∼65% reduction in *prel-3* mRNA levels, respectively (Figure 3E). These data suggest that intact mitochondrial phospholipid trafficking is necessary not only for development but also for maintaining lifespan, aligning with studies demonstrating the importance of mitochondrial membrane composition in ageing and health span regulation (Medkour et al., 2017; Sharma et al., 2019).

### 3.5 PREL-1 and PREL-3 maintain mitochondrial phospholipid homeostasis and respiratory function

**FIGURE 4:**
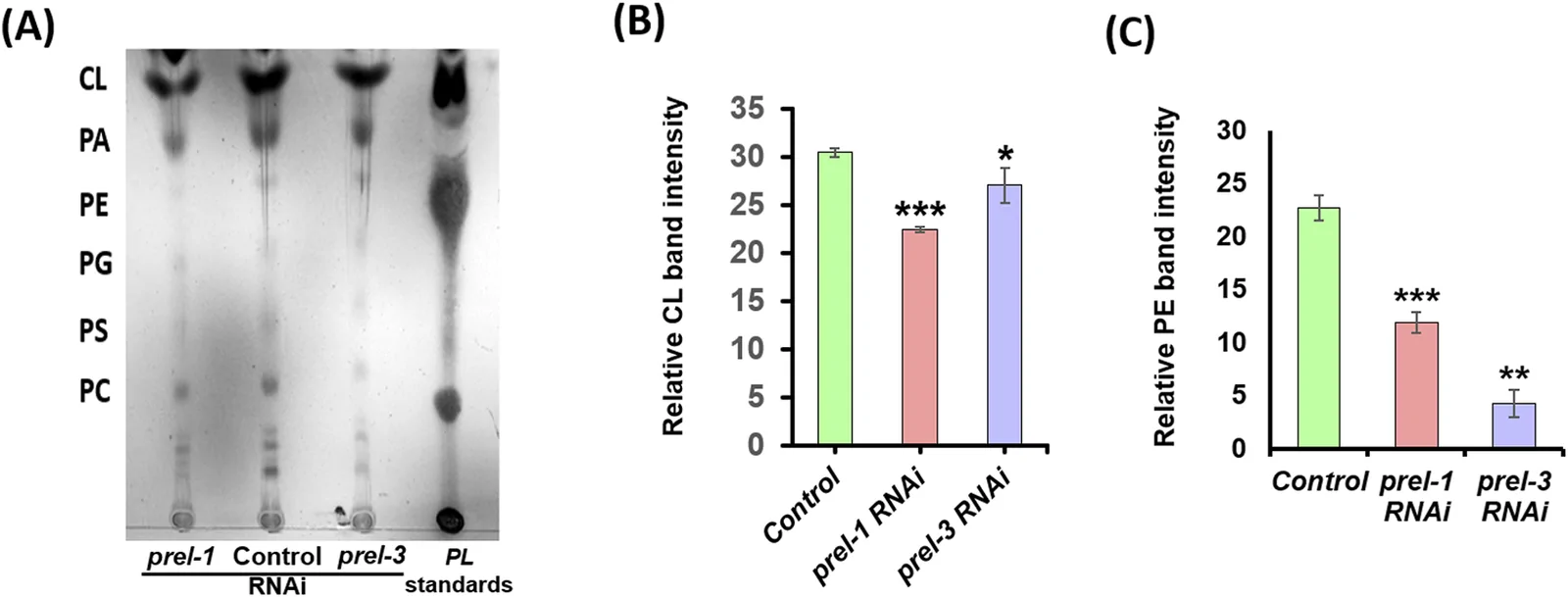
Thin-layer chromatography separation of mitochondrial phospholipids and quantification. **(A)** Thin-layer chromatography for phospholipid separation. Lane1-mitochondrial phospholipids extracted from *prel*-1 RNAi worms, Lane 2-mitochondrial phospholipids extracted from control worms, Lane 3- mitochondrial phospholipids extracted from prel-3 RNAi worms, and Lane 4-phospholipid standards. **(B)** Densitometric analysis of CL bands using ImageJ. Error bars represent mean ± SEM (*p < 0.05, **p < 0.01 and ***p < 0.001). **(C)** Densitometric analysis of PE bands using ImageJ. Error bars represent mean ± SEM (*p < 0.05, **p < 0.01 and ***p < 0.001).

**FIGURE 5:**
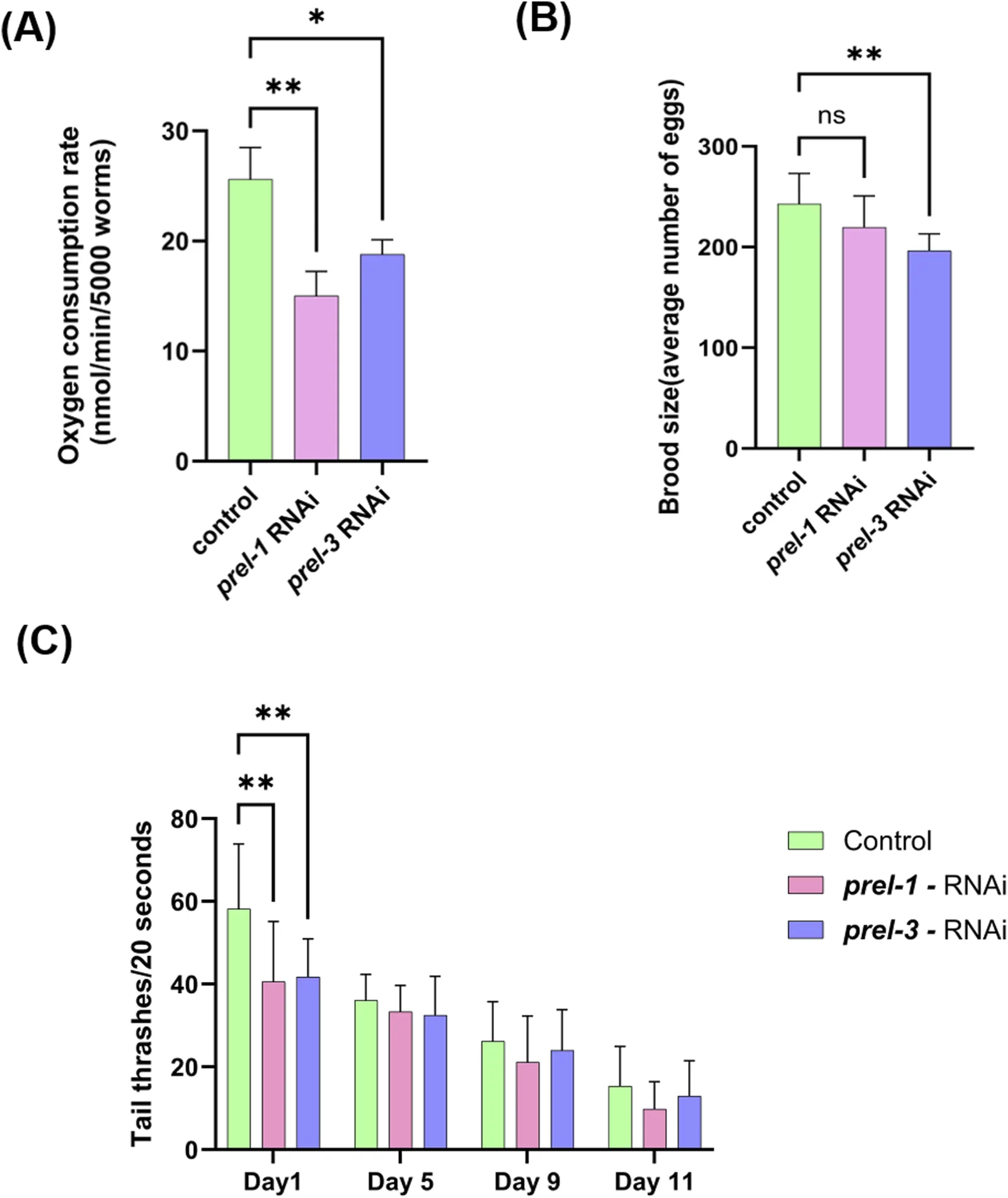
Knockdown of PREL-1 and PREL-3 physiologically affects respiration, reproduction, and motility in *C. elegans*. **(A)** Oxygen consumption rate (OCR) measured in control and RNAi-treated adult worms, expressed as nmol O_2_/min/5000 worms. Data represent three independent experiments and are presented as mean ± SEM (*p < 0.05, **p < 0.01, one-way ANOVA). **(B)** Average brood size of control and RNAi-treated adult worms from day one of adulthood, indicating reproductive output following *prel*-1 and *prel*-3 knockdown (n = 30 worms per condition). Data are presented as mean ± SEM (ns = not significant, **p < 0.01, one-way ANOVA). **(C)** Tail-thrashing assay used as a proxy for motility in control and RNAi-treated adult worms, measured on Days 1, 5, 9, and 11 of adulthood (n = 15 worms per condition). Data are presented as mean ± SEM (**p < 0.01, two-way ANOVA with Tukey’s post-hoc correction).

Given that PA and PS serve as precursors for CL and PE synthesis at the IMM, we predicted that disrupting their transport would alter mitochondrial phospholipid composition. Phospholipid standards were used to standardize TLC runs using the mobile and stationary phases mentioned in the materials and methods (Supplementary Figure 5). Consistent with this hypothesis, mitochondrial lipid profiling revealed distinct phospholipid defects in RNAi-treated worms. To ascertain that the isolated mitochondria was of desirable purity we checked the CL levels from whole cell, cytoplasmic and mitochondrial fractions of control worms (Supplementary Figure 6A) Loss of PREL-1 resulted in a significant reduction in CL levels (Figures 4A,B; p = 0.0021), whereas depletion of PREL-3 decreased mitochondrial PE content (Figures 4A,C; p = 0.0246). The partial nature of these reductions likely reflects incomplete knockdown by the RNAi method and the presence of compensatory or alternative lipid transport pathways, as suggested in other systems (Connerth et al., 2012). To distinguish whether the observed perturbations in the mitochondrial phospholipid levels was because of phospholipid transport to mitochondria or a consequence of phospholipid biosynthesis, degradation, or remodelling, we probed the whole cell phospholipid levels of control and mutant worms. Our results showed approximately equal amounts of whole cell phospholipids under all conditions (Supplementary Figure 6B). This result suggests that the observed reduction in mitochondrial phospholipid levels is most likely because of decreased mitochondrial phospholipid transport. Taken together, these findings suggest an *in vivo* role of PRELI-domain proteins regulating distinct mitochondrial phospholipid pools in *C. elegans*. In line with these observations, oxygen consumption rates were significantly decreased in knockdown worms (Control vs. *prel-1* RNAi p = 0.0029; control vs. *prel-3* RNAi p = 0.0238) (Figure 5A) as compared to the controls (original traces computed by the oxytherm respirometer has been added as Supplementary Figure 7) Reduced respiration likely reflects dysfunction of electron transport chain proteins and impaired mitochondria dynamics, phenomena previously associated with phospholipid imbalance.

### 3.6 Disruption of mitochondrial phospholipid homeostasis impairs organismal fitness and induces oxidative stress

Mitochondrial dysfunction often manifests as systemic physiological impairment. To examine the physiological consequences of mitochondrial phospholipid imbalance, we assessed locomotion, reproduction, stress signalling and ROS accumulation in RNAi-treated worms. Reproductive output was diminished, as evidenced by reduced brood size in PREL-3 (control vs. *prel- 3* RNAi p = 0.0012) knockdown conditions as compared to control worms (Figure 5B). PREL-1 knockdown did not show significant decrease in brood size compared to control (p = 0.1141) (Figure 5B). Locomotor activity assessed by thrashing assays was also reduced in RNAi-treated worms, with early onset (day 1 of adulthood) (control vs. *prel-1* RNAi p = 0.0067 and control vs. prel-3 RNAi p = 0.0036) (Figure 5C). Surprisingly, knockdown did not show any significance in locomotion throughout the adulthood. Collectively, these pleiotropic defects demonstrate that disruption of mitochondrial phospholipid homeostasis affects neuromuscular function, reproduction, and overall fitness in the *C. elegans* model system.

**FIGURE 6:**
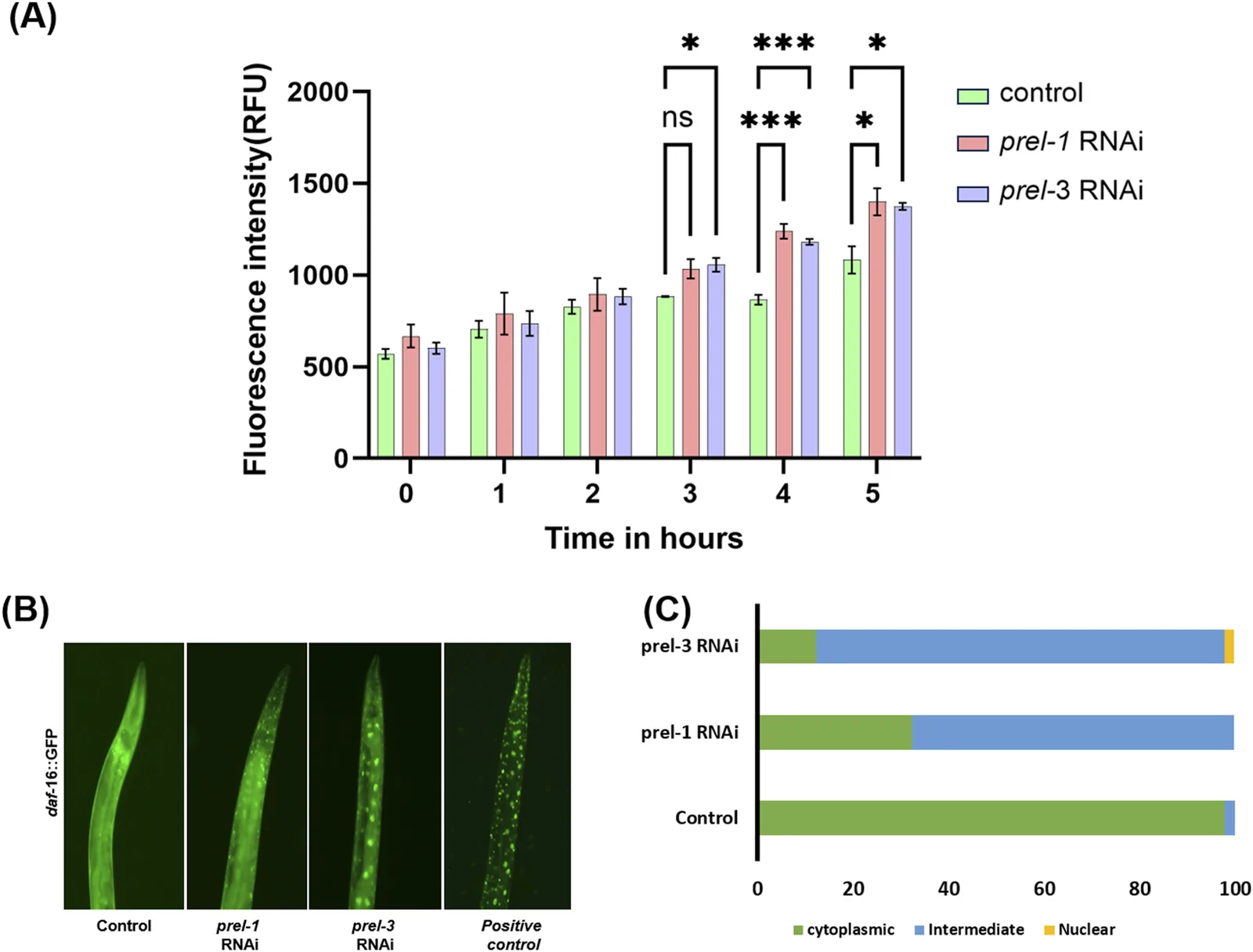
Mitochondrial phospholipid imbalance causes DAF-16 nuclear localization and ROS accumulation. **(A)** Reactive oxygen species (ROS) levels in control and mutant worms, quantified as fluorescence intensity (n = 100 worms per condition). Data represent mean ± SEM; statistical significance was determined by one-way ANOVA with Dunnett's multiple comparisons test (*p < 0.05, **p < 0.01, ***p < 0.001, ****p < 0.0001 vs. control). **(B)** Representative images of DAF-16::GFP localization in wild-type, *prel*-1 RNAi, and *prel*-3 RNAi worms. Heat-stressed, age-synchronized wild-type worms were used as a positive control for nuclear translocation (n = 30 worms per condition). **(C)** Quantification of DAF-16::GFP localization (cytoplasmic, intermediate, or nuclear) is shown as a percentage of total worms scored. Data represent mean ± SEM from three independent trials (n = 30 worms/trial; N = 90 total per condition). Statistical significance was determined by one-way ANOVA with Dunnett's multiple comparisons test (*p < 0.05, **p < 0.01, ***p < 0.001, ****p < 0.0001 vs. control).

Since mitochondrial dysfunction is associated with oxidative stress, we analysed ROS dynamics and *daf*-16::GFP localization. Measurement of cellular ROS levels revealed distinct temporal patterns. PREL-1 knockdown resulted in a significant increase in ROS levels emerging from the 3-h timepoint onward and sustained thereafter (p = 0.0029) (Figure 6A). PREL-3 depletion led to a delayed but pronounced ROS surge beginning at 3 h time point (p = 0.0238) (Figure 6A). These findings indicate that mitochondrial lipid imbalance induces oxidative stress, which may contribute to the observed physiological decline and altered stress-response signalling. We also examined *daf*-16::GFP localisation. DAF-16 is a major insulin/IGF-1 signalling–mediated stress response pathway in *C. elegans*. DAF-16 is a transcription factor that localizes from the cytoplasm to the nucleus under stress conditions. Under control conditions, *daf*-16::GFP predominantly showed cytoplasmic localisation. In contrast, RNAi-treated worms showed nuclear localization (Figures 6B,C), suggesting altered DAF16/FOXO signalling and intense stress response.

These findings indicate that mitochondrial lipid imbalance induces oxidative stress, which may contribute to the observed physiological decline and altered stress-response signalling.

## 4 Discussion

Mitochondrial phospholipids are increasingly recognised as active regulators of organelle architecture, bioenergetics, and signalling rather than passive structural components (Dudek, 2017). While the biosynthetic pathways for cardiolipin (CL) and phosphatidylethanolamine (PE) are well described, the mechanisms by which their lipid precursors are transported across mitochondrial membranes, phospholipids regulation, and their influence on the overall wellbeing of the organism remain poorly understood in a multicellular model organism. In this study, we identify two PRELI-domain proteins in *C. elegans*, B0334.4 (PREL-1) and F15D3.6 (PREL-3), and provide converging computational, biochemical, and physiological evidence consistent with a role as putative intramitochondrial phospholipid transporters with distinct substrate specificities. It should be noted that, while conserved PRELI-family members in yeast and mammals have been directly shown to transfer phospholipids in reconstituted systems, our present data based on homology modelling, docking, RNAi phenotypes, and organelle lipid profiling support but do not directly demonstrate lipid transfer activity by PREL-1 and PREL-3 themselves; direct biochemical transport assays (e.g., liposome-based transfer assays) will be required to formally establish this function.

*In silico* analyses revealed that PREL-1 preferentially binds phosphatidic acid (PA) compared to PREL-3. Our findings demonstrate that the phospholipid transport system plays a critical physiological role in a multicellular organism. While yeast studies have largely focused on biochemical reconstitution and mitochondrial lipid composition (Miliara et al., 2019; Lu et al., 2020), our *in vivo* data show that disrupting these transport steps has profound consequences on development (Figures 3A–C), lifespan (Figure 3D), and stress dynamics in *C. elegans* (Figures 6A–C). This highlights that mitochondrial phospholipid transport is not merely required for organelle maintenance but is tightly coupled to organismal physiology.

CL and PE are known to play non-redundant roles in shaping inner mitochondrial membrane curvature, stabilising respiratory chain super-complexes, and maintaining membrane potential (Gohil et al., 2004; Chan and McQuibban, 2012). It should be noted that the incomplete loss of CL and PE likely reflects the partial nature of RNAi-mediated knockdown and/or the existence of compensatory lipid transport or remodelling pathways. For example, in studies involving yeast cells, knockout of UPS1 or UPS2 did not correlate with complete loss of CL and PE, respectively. Additionally, the loss of mitochondrial phospholipids could be due to an overall decrease in cellular phospholipids resulting from increased degradation, decreased biosynthesis, or altered remodelling of CL. Our results with whole cell phospholipid levels clearly showed that the cellular phospholipids remained unaltered with a concomitant change in mitochondrial phospholipids in mutant cells. Similar redundancy has been proposed in other organisms, in which an alternative lipid transport system may partially buffer the loss of individual transporters (Aaltonen et al., 2016). This buffering may explain why knockdown animals remain viable while exhibiting progressive physiological decline rather than acute lethality.

One of the most striking aspects of our study is the breadth of physiological defects arising from disruption of the mitochondrial phospholipid homeostasis. Impaired development, shortened lifespan, altered lipid metabolism, decreased locomotion, and diminished reproductive output collectively point to a systemic collapse in metabolic robustness. Similarly, impaired locomotion likely reflects energy deficits arising from defective oxidative phosphorylation. Surprisingly, in the present study, mitochondrial defects accompanied by reduced respiratory capacity were associated with decreased organismal lipid accumulation, possibly suggesting metabolic reprogramming toward enhanced lipid mobilisation or reduced lipid biosynthesis (Hardie, 2011; Longo et al., 2016). As central regulators of energy homeostasis, mitochondria control lipid metabolism through ATP production, acetyl-CoA availability, and β-oxidation. Reduced mitochondrial respiration may activate energy stress pathways that promote lipid catabolism and suppress lipid synthesis, thereby lowering lipid storage. Together, these findings indicate that mitochondrial dysfunction drives compensatory metabolic adaptation that reshapes organismal lipid homeostasis.

Our ROS measurements reveal distinct kinetics of oxidative stress induction following depletion of PREL-1 and PREL-3. The rapid ROS accumulation in PREL-1 knockdown worms may reflect acute destabilisation of CL-dependent respiratory complexes, which are particularly sensitive to CL loss (Petit et al., 2020). In contrast, the delayed ROS surge in PREL-3-depleted animals suggests a more gradual impairment of mitochondrial function, potentially linked to altered PE-dependent membrane dynamics.

Interestingly, both knockdowns were associated with nuclear localization of *daf-16*::GFP fluorescence. This indicates that mitochondrial phospholipid imbalance may induce a coordinated stress response characterised by activation of *Daf-16*/FOXO signalling, thereby enhancing protective transcriptional programs that support stress adaptation and mitochondrial maintenance (Durieux et al., 2011; Cheng et al., 2025).

Defects in mitochondrial phospholipid metabolism are increasingly linked to human pathologies, including Barth syndrome, neurodegenerative disorders, and metabolic diseases (Clarke et al., 2013; Lamari et al., 2013; Sergi, 2025; Wang et al., 2025). By identifying discrete transport steps that connect lipid precursors at the outer mitochondrial membrane to phospholipid synthesis at the inner membrane, our study provides a mechanistic framework that may help explain how subtle alterations in lipid trafficking can lead to profound cellular and physiological consequences. Importantly, *C. elegans* provides a powerful platform for dissecting these processes *in vivo*, enabling direct linkage between molecular defects and organismal phenotypes. The conservation observed here suggests that insights gained from this model are likely to be broadly relevant across metazoans.

A key limitation of this study is the use of RNAi, which results in partial loss of function. Complete genetic ablation of PREL-1 or PREL-3 proteins may reveal more severe phenotypes or lethality and could clarify the full extent of their physiological roles. Because specific antibodies against PREL-1 and PREL-3 are not available, the present study only evaluated gene downregulation at the transcript level. Although the increased levels of CL in mitochondrial fraction as compared to other cellular fractions indicate adequate mitochondrial enrichment (Supplementary Figure 6A), it should be noted that a protein-based (Western blot) marker assay for endoplasmic reticulum, cell membrane and vacuolar contamination detection would further strengthen confidence in mitochondrial purity. Another limitation of the study was not to test any phospholipid biosynthetic or remodelling pathways for mitochondrial phospholipid perturbations or not performing any radiotracer-based pulse chase experiments. However, we performed whole cell vs. mitochondrial phospholipid distribution measurements in wildtype and mutant worms to address these issues. Moreover, it should be emphasized that a LC-MS/MS-based lipidomic study to measure mitochondrial phospholipid content of these mutant worms would definitively provide evidence that PREL-1 and PREL-3 are indeed phospholipid transport proteins. Additionally, identifying interacting partners and alternative lipid transport pathways will be essential to understanding how mitochondrial lipid homeostasis is maintained under stress. Future work could also explore whether pharmacological modulation of lipid composition can rescue mitochondrial defects in these models, providing a potential therapeutic avenue for mitochondrial lipid disorders.

In summary, this study provides converging computational and physiological evidence that PREL-1 and PREL-3 act as putative intramitochondrial phospholipid transporters in *C. elegans*. Our findings demonstrate that mitochondrial phospholipid homeostasis is a critical determinant of mitochondrial function, organismal development, ageing, and metabolic health (Graphical abstract). By bridging molecular lipid trafficking with whole-organism physiology, this work expands our understanding of mitochondrial lipid biology and highlights new avenues for investigating mitochondrial dysfunction in health and disease.

## Data Availability

The data presented in the study are deposited in the GitHub repository, with accession link: https://github.com/skirut/PRELI_Computational_Pipeline.
