# Supplementary material for: Functional characterisation of PREL-1 and PREL-3 proteins as putative intramitochondrial phospholipid transporters governing homeostasis and organismal health in Caenorhabditis elegans
Source: Front Mol Biosci. 2026 Aug 19;13:1892600. doi: 10.3389/fmolb.2026.1892600 (PMC13534800; doi:10.3389/fmolb.2026.1892600)
Supplement: Supplementary file 1 [file DataSheet1.docx]

**Functional characterisation of PREL-1 and PREL-3 as intramitochondrial phospholipid transporters governing homeostasis and organismal health in *Caenorhabditis elegans.***

**Supplementary Figures:**

**Supplementary Figure 1.** **Comparative sequence identity and similarity analysis of C. elegans PREL-1 with PRELI-family homologues using multiple substitution matrices.**

(A) Heatmaps represent sequence similarity calculated using multiple amino acid substitution matrices, including BLOSUM and PAM matrices.The colour gradient represents the percentage of sequence conservation between PREL-1 and homologous PRELI-family proteins, with higher values indicating greater evolutionary conservation.
(B) Heatmaps represent sequence identity values calculated using multiple amino acid substitution matrices, including BLOSUM and PAM matrices.The colour gradient represents the percentage of sequence conservation between PREL-1 and homologous PRELI-family proteins, with higher values indicating greater evolutionary conservation.

**Supplementary Figure 2.**  **Comparative sequence identity and similarity analysis of C. elegans PREL-3 with PRELI-family homologues using multiple substitution matrices.**

(A) Pairwise sequence comparison of C. elegans PREL-3 with PRELI-domain-containing homologues from human, yeast, and other representative species. Heatmaps represent sequence similarity calculated across different substitution matrices (BLOSUM and PAM).The colour scale indicates relative conservation levels, where higher values represent increased amino acid similarity or identity between compared sequences.
(B) Pairwise sequence comparison of C. elegans PREL-3 with PRELI-domain-containing homologues from human, yeast, and other representative species. Heatmaps represent sequence identity calculated across different substitution matrices (BLOSUM and PAM).The colour scale indicates relative conservation levels, where higher values represent increased amino acid similarity or identity between compared sequences.

**Supplementary figure 3:**

(A) Predicted docking conformation of phosphatidic acid (PA) within the conserved PRELI-domain lipid-binding cavity of the C. elegans PREL-3 structural model. PREL-3 is represented using cartoon and molecular surface visualization (blue), while PA is shown as a red stick representation. The magnified binding-pocket view highlights the ligand orientation and surrounding amino acid residues predicted to contribute to phospholipid stabilization.
(B) Two-dimensional protein–ligand interaction map of the PREL-3–PA docking complex illustrating amino acid residues contributing to ligand stabilization through hydrophobic, hydrogen-bonding, electrostatic, and aromatic interactions. Interaction types and residue chemical classifications are indicated according to the colour scheme shown below the interaction maps.

**Supplementary Figure 4. Ethanolamine supplementation rescues the developmental delay caused by *prel-3* knockdown**

(A) Representative brightfield images showing developmental progression of control, control + ethanolamine (Etn), *prel-1* RNAi, *prel-1* RNAi + Etn, *prel-3* RNAi, and *prel-3* RNAi + Etn worms at 0, 12, 24, and 36 hours post-synchronization. (B) Body size quantification at 24 hours showing partial rescue of the *prel-1* RNAi developmental delay by ethanolamine supplementation. (C) Body size quantification at 24 hours showing rescue of the *prel-3* RNAi developmental delay by ethanolamine supplementation. (D) Body size quantification at 36 hours shows no significant rescue of the *prel-1* RNAi developmental delay by ethanolamine supplementation. (E) Body size quantification at 36 hours showing rescue of the *prel-3* RNAi developmental delay by ethanolamine supplementation. For (B–E), data represent mean body length (μm) ± SEM (n = [20] worms per condition, per group). Statistical significance was determined by one-way ANOVA with Tukey's test; only the comparison between RNAi and RNAi + Etn is shown for clarity (ns, not significant; *p < 0.05; ****p < 0.0001).

**Supplementary image 5: Thin-layer chromatography (TLC) separation of phospholipid standards**

Lanes correspond to individual phospholipid standards: phosphatidylcholine (PC), phosphatidylserine (PS), phosphatidylglycerol (PG), phosphatidylethanolamine (PE), phosphatidic acid (PA), and cardiolipin (CL). Following development, lipid spots were visualized by cupric sulphate staining.

**Supplementary image 6: TLC validation**

**(A)** Total lipids were extracted from whole-cell lysates, cytoplasmic fractions, and mitochondrial fractions of *C. elegans* and resolved by thin-layer chromatography to compare phospholipid distribution across cellular compartments. A distinct band corresponding to cardiolipin (CL) is enriched in the mitochondrial fraction relative to the whole-cell and cytoplasmic fractions, consistent with CL's known localization to the inner mitochondrial membrane and confirming the purity and mitochondrial identity of the isolated fraction.

**(B)** Whole-cell phospholipid profiles of control, *prel-3* RNAi, and *prel-1* RNAi worms.

**Supplementary Figure 7: Representative OxyTrace graphs of control, *prel-1* RNAi and *prel-3* RNAi**

Representative traces of oxygen level plotted over time (min) recorded using the OxyTrace system for worms subjected to control (Ht115), *prel-1* RNAi, or *prel-3* RNAi treatment (top to bottom panels, respectively). Each trace reflects the oscillatory pattern of oxygen consumption.
